# Cancer Care in the United States: A System in Transition

**Published:** 2015-07-01

**Authors:** Pamela Hallquist Viale

The American Society of Clinical Oncology (ASCO) published its State of Cancer Care in America report for 2015 in the March issue of the *Journal of Oncology Practice*. The report contains many important facts about the cancer care system in the United States and focuses on several key issues. Significant improvements were made, including longer 5-year survival rates for many cancers and the approval of 10 new drugs and new tests, which demonstrate progress in the way we care for our patients with cancer. I urge you to read the report in its entirety. For those of you who cannot get to it right away, here’s a snapshot of the highlights in the report.

## CHANGES IN CANCER CARE IN AMERICA

Our population is aging and expanding, requiring more services for cancer care (ASCO, 2015). The Affordable Care Act (ACA) increased the number of Americans with insurance, contributing to the demand for cancer care. Increased numbers of survivors requiring long-term care and an aging population add up to a need for more cancer care services. And although the ACA has certainly benefited many individuals, advances in cancer screening and treatment have not occurred across all racial and ethnic groups, creating a disparity in cancer care services.

## NEW CONCERNS FOR PUBLIC HEALTH

The ASCO report cites two new issues affecting the need for cancer care services: obesity and electronic cigarettes. Obesity may lead to approximately eight types of cancer and can affect survival from cancer as well. With more than one-third of adults in the United States today considered obese (and one-fifth of our children), we can’t afford not to educate the public about this modifiable risk factor (ASCO, 2015). And although electronic cigarettes have been touted as a viable option to cigarette smoking, evidence does not support that conclusion.

## THE ONCOLOGY WORKFORCE

Much has been written about the projected deficit between the number of oncologists currently and the number needed to meet the needs of cancer care services. ASCO reports that more than 18,000 physicians provide oncology subspecialty care. But the aging workforce (there are more oncologists aged 65 and older compared with those entering the field) is a major concern. Burnout and a lack of services in rural settings continue to be problems; the number of providers interested in private or solo practice continues to decline.

You might wonder whether advanced practitioners (APs) were mentioned in the ASCO report. I’m pleased to say that APs were included in the provider section, with the report noting that more than 3,000 APs were providing cancer care across the country, including nurse practitioners (NPs), doctors of nursing practice, and physician assistants (PAs). The authors of the report also described the employment of APs as growing rapidly and noted that these increased numbers could make a difference in the number of providers who might decide to enter a career in oncology.

The ASCO State of Cancer Care in America report also noted that with the increased focus on value and quality of care by patients and payers alike, a team-based care approach has taken a heightened role, enabling clinicians to improve care and help to reduce workforce shortages. The report describes NPs as having chemotherapy-prescribing authority and notes that in 20 states, individuals can practice as independent providers with no limitations on scope of practice (ASCO, 2015). Approximately 1,900 NPs are certified in oncology, and approximately 1,800 PAs are working in oncology.

Encouragingly, the US Bureau of Labor Statistics anticipates that employment of NPs and PAs will increase by 31% and 38.4%, respectively, by the year 2022, a statistic that is much greater than the projected increase in numbers of physicians and surgeons (17.8%; US Department of Labor, 2015a, 2015b). The ASCO 2014 census of US oncology practices reported that there are a large number of practices employing APs (more than 2,700 NPs and 1,100 PAs), with most working in academic centers (ASCO, 2015).

## ASCO REPORT CONCLUSION

Continued challenges include fragmentation of cancer care across many providers and settings, cost of care, barriers to access, difficult payment structures, cancer drug shortages, and a lack of clarity regarding treatment goals (ASCO, 2015). ASCO’s strategies focus on the use of innovative payment models, the above-mentioned expansion of team-based care, shared decision-making, and initiatives to target the rural care setting.

The current state of cancer care in the United States is in transition, with significant changes in the site of practice, new care-delivery models, and the increasing concern regarding the escalating costs of cancer care. The encouraging trends of increased survival rates, new treatments, and new methods of health-care delivery point to a positive outlook for our patients with cancer (ASCO, 2015). I am especially encouraged not only by the fact that APs are an integral part of the assessment of the workforce in a report on cancer care, but that we are considered part of the solution to the problems noted in the report. Advanced practitioners working in oncology today have a visible and increasingly important role in cancer care.

If you are interested in more information on the 2015 ASCO State of Cancer Care in America report, you can read the full text in the *Journal of Oncology Practice* or read the Executive Summary available at http://www.asco.org/practice-research/cancer-care-america-2015/welcome.

## References

[1] (2015). The state of cancer care in america, 2015: a report by the american society of clinical oncology.. *Journal of oncology practice / American Society of Clinical Oncology*.

[2] (2015a). Occupational Outlook Handbook​.​ Physician ​a​ssistants​ (2014-​20​15 ​ed.).. US Department of Labor. (2015a). ​Bureau of Labor Statistics, US Department of Labor​.​.

[3] Occupational Outlook Handbook​.​ Nurse ​a​nesthetists, ​n​urse ​m​idwives, and ​n​urse ​practitioners​ (2014-​20​15 ​ed.). US Department of Labor. (2015​b​).​ ​Bureau of Labor Statistics, US Department of Labor​..

